# Low Caffeine Concentrations Induce Callus and Direct Organogenesis in Tissue Cultures of *Ornithogalum dubium*

**DOI:** 10.3390/plants14071127

**Published:** 2025-04-05

**Authors:** Carloalberto Petti

**Affiliations:** Department of Applied Science, EnviroCORE, South East Technological University, Kilkenny Road, R93V960 Carlow, Ireland; carloalberto.petti@setu.ie

**Keywords:** *Ornithogalum* spp., micropropagation, caffeine, phytohormone, phloroglucinol

## Abstract

Caffeine is a nitrogenous base that naturally occurs in coffee (*Cafea arabica*), tea (*Thea sinensis*), and cocoa (*Theobroma cacao*). Chemically, caffeine is 1,3,5-trimethylxanthine, a purine analogue. Due to significant human consumption, caffeine effects have been widely studied. Being a natural xanthine derivative, the key degradative enzyme is xanthine oxidase, converting caffeine into 1-methyluric acid. Ecologically, caffeine is believed to act as a repellent molecule against insect feeding behavior. Caffeine’s chemical similarity to purines and plant hormones motivated this study, establishing the potential for cellular de-differentiation and re-differentiation. For this, a highly hormone-responsive plant species, *Ornithogalum dubium*, was used. As caffeine has been shown to induce endoreplication, the potential for new germlines in *O. dubium* is attractive. Using tissue culture, a range of caffeine concentrations were used (0.0125 mg/L to 2.0 mg/L) without additional hormones. A significant difference (*p* > 0.05) was observed for intermediate concentrations of 0.0125, 0.025, and 0.05 mg/L when compared to the control (no hormones). The highest rates of callus induction were obtained at a concentration of 0.025 mg/mL. Higher concentrations were phytotoxic (1.0 mg/L or greater). To conclude, caffeine-regenerated plants were not dissimilar to those obtained from canonical hormones.

## 1. Introduction

**Figure 1:**
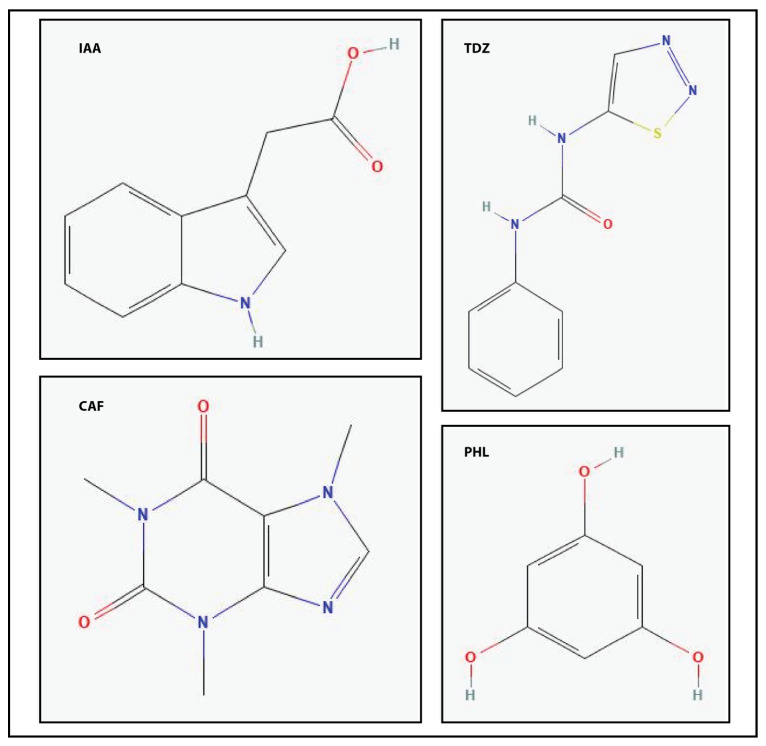
Chemical structure of reference hormones and non-typical hormone-like acting substances. The image shows the structure of indole acetic acid (IAA), a naturally occurring auxin; Thidiazuron (TDZ), a synthetic cytokinin; caffeine, a naturally occurring nitrogenous base; and phloroglucinol, a hormone-like phenolic compound.

Caffeine, the main metabolite found in coffee, *Coffea species*, and tea, *Camellia sinensis*, is an alkaloid with a structure like a purine; chemically, it is 1,3,5-trimethylxanthine (Figure 1). Purine alkaloids are widely distributed in the plant kingdom, and caffeine is found widespread in a variety of plant species, including *Citrus* species; flowers and pollen, such as the seeds of *Cola nidica*; guarana (*Paulliana cupana*); maté (*Ilex paragiariensis*); and cocoa (*Teobroma cacao*) [1]. Caffeine has also been identified in the sclerotia of the fungal species *Claviceps sorhicola* [2,3]. The abundance of caffeine in dedicated plant tissues, at times as high as 2%, can be interpreted via two hypotheses. The chemical defense theory suggests that caffeine contributes to the protection of flowers, fruits, and young leaves against phytophagous insect larvae and insects [4,5,6,7]. The alternative/complementary hypothesis, the allelopathic theory, speculates that caffeine in seed coats can be released into the soil and impede/prevent other seeds from germinating [8,9,10,11,12,13].

While caffeine can be found in as many as 100 species, mostly associated with dicots, rare exceptions occur in monocots such as *Scilla marittima* [1]. The pathway to its formation is predominantly understood in the species *Coffea* and *Camellia*, that is, coffee and tea [1,14]. Xanthosine serves as the precursor, which can be used through four possible pathways, the *de novo* route, the adenosine monophosphate- AMP route, the S-adenosylmethionine- SAM cyclic route, and the Guanosine-monophosphate GMP route, with the most important routes being the production of xanthosine via inosine-5’ monophosphate, the latter derived from *de novo* purine nucleotide biosynthesis, and the pathway in which adenosine is released from S-adenosyl l-homocysteine is then converted into xanthosine [1]. Huang and collaborators [15] demonstrated that the pathway to caffeine has evolved independently multiple times in different species through convergent evolution.

Extensive research has taken place in humans in relation to the effects of caffeine, positive or negative, due to the vast use of coffee and tea throughout the world (e.g., see [16,17,18]). In plants, caffeine has been investigated mainly for its potential antimicrobial properties [19,20,21,22]. Moreover, the use of transgenic plants expressing caffeine has shown promise in the control of fungal and bacterial pathogens [23,24,25,26]. In micropropagation studies, caffeine is phytotoxic when used at high concentrations (1%, [27]) and induces polyploidization when used at lower concentrations, but it is species-dependent. For instance, Roy [27] showed that little to no chromosomal variation occurred when diluted sub-lethal concentrations of caffeine were used (0.25–0.5%), whereas some polyploidization events took place at a concentration of 0.1% on the nodal roots of *Callisia fragrans*. Moreover, chromosome duplication occurs at a concentration of 0.3% in *Lilium* [28]. Conversely, in wheat, efficient rates of chromosome duplication, albeit non-significant, have been seen taking place at 0.5 mM (or 0.1%) [29]. In a recent report by Muratova and collaborators [30], caffeine was used in micropropagation experiments with *Rubus sp.*, showing that caffeine was able to increase rhizogenesis rates. This effect was more pronounced when the medium contained auxins. At a concentration above 100 mg/L, phytotoxic effects were evident. Overall, the literature evidenced that there are a limited number of studies related to caffeine and tissue cultures in plants.

*Ornithogalum dubium* is an attractive bulbous plant belonging to the Hyacinthaceae family. The genus is widespread, with more than 120 species distributed across Africa, Europe, and Western Asia. The exact number of species is still a matter of debate, with current estimates at 160 species [31]. *Ornithogalum dubium*, among other species, has found its way in cultivation as a valued ornamental plant for both cut flowers and potted plants [32]. Its original flower’s color, orange with a dark internal grey/black core, has been enriched with yellow and white through hybridization with other species, thereby increasing the commercial appeal of the species [33]. The species is commonly found in South Africa, living on mountain slopes and flats in stony clay soils, and it is not considered threatened.

*Ornithogalum dubium* has been utilized for cut flowers, with extensive crossbreeding conducted to enhance the color range and height of the inflorescence [33,34,35]. Cross-species hybridizations, even among species that are highly morphologically similar, are often unsuccessful due to genetic barriers, and sophisticated embryo rescue techniques are required to enhance the success of such hybridizations [36]. Recently, *O. dubium* has attracted significant interest as a winter-flowering bulb, with shorter height varieties being selected [37]. Storage and glasshouse conditions are important factors in timing the flowering stage [38]. A number of pathogens can affect *Ornithogalum,* with *O. dubium* being particularly sensitive to soft rot caused by *Erwinia carotovorum*. Recent efforts in breeding have developed promising new stocks with enhanced resistance to this pathogen [39]. Increased resistance to soft rot has also been achieved through genetic engineering by expressing a small antimicrobial peptide from the Asian horseshoe crab, tachyplesin [40]. *Ornithogalum dubium* has been amenable to transformation via both ballistic and *Agrobacterium* methods [41,42,43], with ongoing improvements to the *Agrobacterium* protocols [44] providing broader opportunities for genetic manipulation. In all cases, though, tissue culture is essential for recovering the desired transformants. *Ornithogalum* has been shown to respond efficiently to micropropagation, with multiple protocols available [45,46,47,48]. Recently, [49] demonstrated a notable plasticity of this species to a variety of hormones and non-canonical compounds, such as Phloroglucinol and elsewhere Benzothiadiazole, BTH, a structural analogue of salicylic acid [50]. This indicates ongoing opportunities to further explore micropropagation in *Ornithogalum* species.

The structural similarity between caffeine and that of cytokinin and indole-3-acetic acid (IAA) compounds, along with the limited availability of micropropagation experiments, prompted this investigation into the potential role of caffeine in the tissue culture of *Ornithogalum dubium*. The main objectives of this study were to assess the potential of caffeine for callus induction, evaluate caffeine phytotoxic levels, assess its impact on the genomic stability of the species, and determine the optimal concentrations for use in tissue culture experiments.

## 2. Results

### 2.1. Chemistry of the Compounds

Chemically, caffeine is 1,3,5-trimethylxanthine, a purine analogue [3]. Its basic structure consists of two rings, which is also common to indole-3-acetic acid (IAA). IAA is also an indole derivative. The caffeine structure contains three additional nitrogen atoms at positions 1, 3, and 7 compared to IAA, with a single nitrogen (Figure 1). Carbon-oxygen double bonds are present in the basic structure, in contrast to the single bond found in the acetyl group side chain of IAA. The structure of thidiazuron (TDZ), a synthetic cytokinin, features two rings: a phenol ring with an amide group that has a ring substituent as a secondary group. Noticeably, the nitrogen-carbonyl-nitrogen group is also present in the caffeine structure. Among the four structures, phloroglucinol—a compound shown to exhibit hormone-like properties [49,51]—is characterized by the simplest structure being a tri-substituted phenolic compound. The similarities between IAA, a canonical auxin; TDZ, a synthetic cytokinin; and caffeine, a purine alkaloid, supported the investigation into caffeine’s potential hormone-like effects.

### 2.2. Evaluation of Caffeine’s Ability to Stimulate Cellular Development in Ornithogalum dubium Tissue Culture

**Figure 2:**
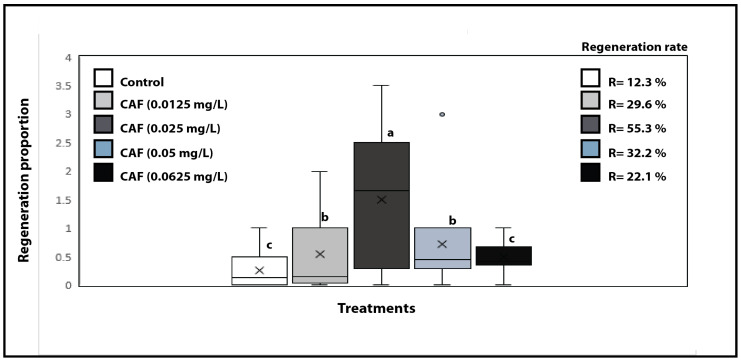
Regeneration proportion of *Ornithogalum dubium* exposed to four concentrations of caffeine. The regeneration proportion was calculated as the ratio of positive fragments to negative fragments for each plate. This figure represents the values of three independent experiments. The x in the box plot indicates the mean value. Different letters denote statistical significance at the α-value of 0.05, while identical letters indicate no significant difference (*p* > 0.05). Regeneration rates are presented in the same figure on the right side of the panel and represent the average of three experiments. Error bars indicate the minimum and maximum values.

The initial set of experiments (*n* = 3) evaluated the potential of caffeine to induce cellular de-differentiation in fragments of *O. dubium*. Two ranges of concentrations were tested: a low concentration range (0.0125, 0.025, 0.05, and 0.0625 mg/L) and a high concentration range (1.0, 1.5, and 2.0 mg/L). At the higher concentrations, caffeine acted as a bleaching agent, resulting in rapid and complete death of the exposed fragments (Supplementary Figure S1), indicating cytotoxicity. In contrast, when caffeine was used at much lower concentrations (1:10 to 1: 80 dilutions), it promoted callus formation across the tested concentrations (Figure 2), with the highest rates determined for 0.025 mg/L (*p* < 0.05). Notably, some background callus formation was observed in the control plates, which contained neither phytohormones nor caffeine. Nonetheless, the rates determined in the control were significantly lower than those for caffeine (Figure 2).

Caffeine induced callus formation in a concentration-dependent manner, ranging from 0.0125 to 0.025 mg/L. However, callus induction rates decreased when tested at concentrations of 0.05 and 0.0625 mg/L, indicating a limited range of activity. The maximum rate of callus formation, determined as the ratio of fragments with callus/microtubers/plantlets to the total number of fragments exposed, was 55.1%. In contrast the lowest rate was observed at the concentration of 0.0625 mg/L which yielded a rate of 22.3%. Variability was noted from plate to plate in the number of fragments that developed calli or exhibited direct organogenesis and those that did not (Figure 2). This variability was also evident in the control group and across all caffeine concentration levels tested; however, the greatest spread was observed at 0.025 mg/L (Figure 2).

### 2.3. Evaluation of Caffeine Compared to Standard Phytohormones

**Figure 3:**
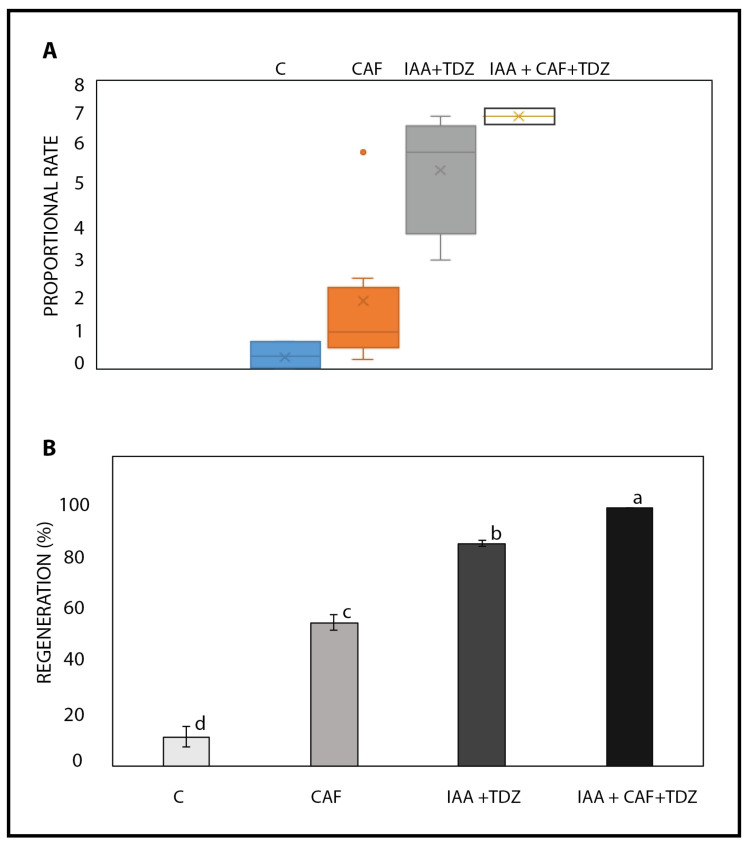
Regeneration rates of *Ornithogalum dubium* exposed to caffeine and the reference hormones IAA+TDZ. In (**A**), regeneration proportions of fragments exposed to no hormones (C), caffeine (0.025 mg/L), IAA+TDZ (0.5/0.5 mg/L), and the combination of IAA and TDZ with caffeine at the same concentrations. The x in the box plot represents the mean value, while the error bars indicate the highest and lowest values. In (**B**), the rates of regeneration resulting from three experiments conducted over a period of 9/12 months are displayed. Different letters denote statistical significance at the α-value of 0.05, while the error bars represent standard deviations. Identical letters indicate no statistical difference (*p* > 0.05).

**Figure 4:**
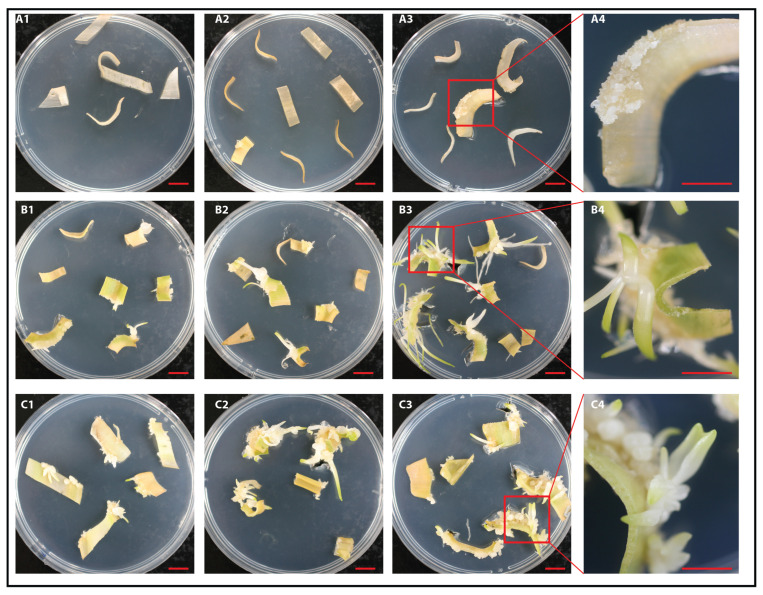
Morphological characteristics of calli/plantlets and microbulbs developed under no hormone (**A1**–**A4**), caffeine ((**B1**–**B4**), 0.025 mg/L), and IAA+TDZ treatments ((**C1**–**C4**), 0.5/0.5 mg/L). The number of fragments per plate varied between 6 and 8, with an average across experiments of 7 fragments. The red bar equates to 0.5 cm. The figure clearly illustrates variability among the plates within each treatment but more prominently in the control (**A1**–**A4**), where callus formation was irregular and occurred infrequently, as well as in the caffeine treatment (**B1**–**B4**).

Petti (2020) [49] demonstrated high rates of callus formation and organogenesis in *Ornithogalum dubium* using various hormones, including IAA and TDZ, as well as a non-canonical compound, phloroglucinol. After establishing that caffeine induces callus formation and direct organogenesis, in a significant manner, we compared its effects to those of IAA and TDZ combination, as well as to the combination of IAA, TDZ, and CAF (Figure 3). Caffeine, at the optimal concentration (0.025 mg/L), consistently demonstrated the ability to induce callus formation in independent experiments (*n* = 3), yielding an average rate of 55.3 ± 9.6% across experiments. Not surprisingly, the combination of IAA and TDZ exhibited a significantly higher rate of 86.2 ± 6.8% (*p* < 0.05) (Figure 3 and Figure 4, C series). When IAA and TDZ were used in conjunction with caffeine at the determined optimal concentration of 0.025 mg/L, the rate increased to 98 ± 5.1% (*p* > 0.05), indicating an additive and/or synergistic effect, although this finding was not statistically significant (*p*>0.05).

The canonical hormones IAA and TDZ were not only more effective in inducing callus formation, but they also demonstrated greater consistency in callus induction, with minimal differences observed among Petri dishes and reduced variability seen at the fragment-to-fragment level (Figure 4, series C). Additionally, callus growth occurred more rapidly than with caffeine (Supplementary Figure S1), generally exhibiting a delay of 1 to 2 weeks between the IAA+TDZ treatment and the CAF treatment.

### 2.4. IAA Inhibitory Studies

When caffeine induction ability was examined for a potential functional relationship with IAA, via the utilization of an IAA inhibitor, L-kynurenine, no differences were observed at any of the tested concentrations. In this study, L-kynurenine was not effective in inhibiting both endogenous auxin levels and exogenously supplied auxin (Supplementary Table S1).

### 2.5. Macro- and Micro-Morphologies

**Figure 5:**
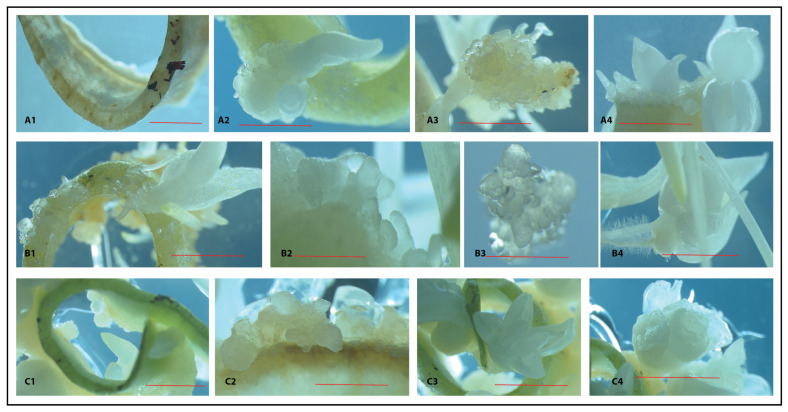
Microphotographs showing various stages of calli/micro-plantlets and micro-bulbs in control fragments (**A1**–**A4**), caffeine exposed fragments (**B1**–**B4**), and in IAA+TDZ exposed fragments (**C1**–**C4**). The red bar equates to 5 mm, except for (**A3**,**B2**,**B3**), where it equals 2 mm.

**Figure 6:**
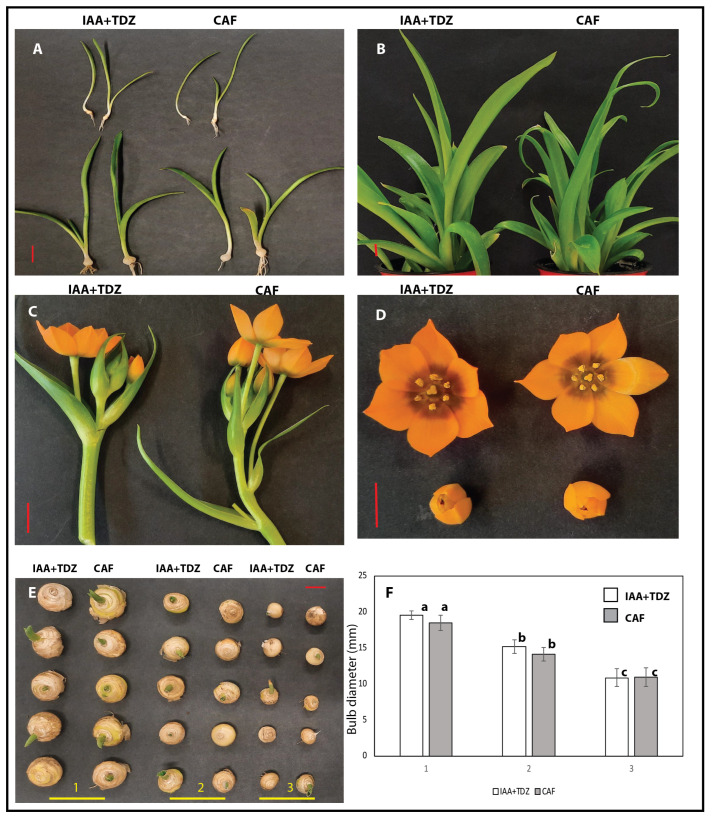
A comparison of caffeine and IAA+TDZ recovered plants through different developmental stages, highlighting a general equivalence between the two treatments. In (**A**), small plantlets arising 1/1.5 months after tissue culture transfer to compost. Panel (**B**) shows well-developed plants of 4/5 months old and approaching the flowering stage. Flowering stage and flower characteristics of representative plants are shown in panels (**C**,**D**). Panel (**E**) presents three classes of bulb sizes from five representative bulbs of each treatment. Panel (**F**) shows the non-statistical difference between the average sizes of the three bulb categories illustrated in figure (**E**). Different letters denote statistical significance at the α-value of 0.05, while the error bars represent standard deviations. Identical letters indicate no statistical difference (*p* > 0.05). The red bars equate to 1 cm.

**Figure 7:**
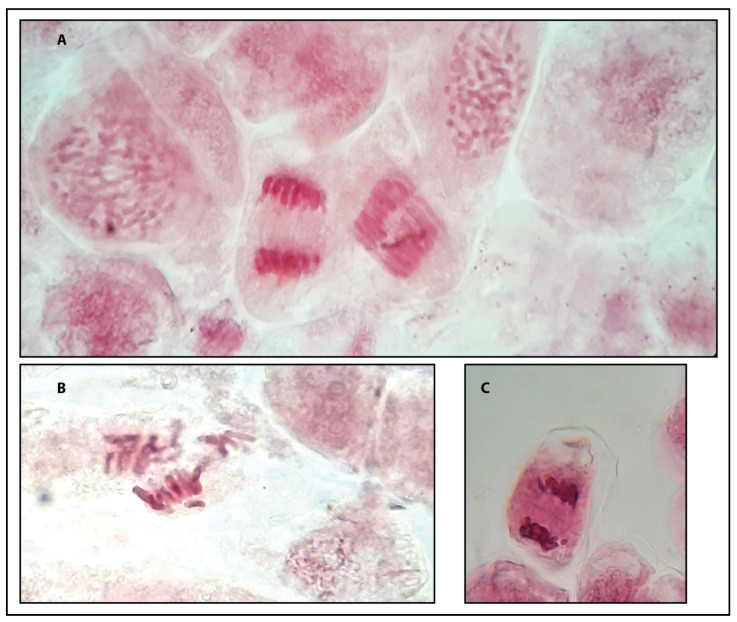
Mitotic stages as observed in CAF-induced callus. In panel (**A**), various clearly visible cells at different stages of prophase and anaphase. In (**B**), metaphase stage (2n = 16), while panel (**C**) depicts late anaphase, showing evidence of the phragmoplast.

The regenerated plants resulting from the caffeine micropropagation experiments, as well as those from the negative and positive control (IAA+TDZ) experiments, were equivalent in all aspects (Figure 5), with no substantial morphological differences observed at the leaf, flower, and bulb levels (Figure 6). Furthermore, no apparent morphological differences were identified in stomata, guard cells, or chromosome numbers (Figure 7), suggesting no direct effect of caffeine on genetic cytotoxicity at the optimal concentration used (0.025 mg/L). To note, though, some variability in the general morphology of certain clonal regenerated plants was observed across all treatments; examples include narrow leaves and variegations. Nevertheless, these occurrences were infrequent, constituting approximately 0.05/0.1/% of the total observations.

## 3. Discussion

In this report, we demonstrated that caffeine could stimulate both cellular de-differentiation and callus formation, as well as indirect and direct organogenesis when used at low concentrations (<1.0 mg/L). The rates determined for caffeine were comparable to those for another non-canonical hormone-like substance, phloroglucinol [49], yet, significantly lower than those for the traditional hormones IAA and TDZ (*p* < 0.05). Notable was the detection of callus formation in the control treatment. This phenomenon was not observed in an earlier study by the same author [49], and it appeared to be quite irregular. This occurrence may be linked to the cultural stage of the experiments and the presence of some endogenous IAA. In all cases, however, the rate of callus induction was significantly lower than that of the caffeine (*p* < 0.05) and IAA+TDZ treatments. Nonetheless, this finding supports the plasticity of *Ornithogalum dubium* in responding to both conventional hormones and non-conventional molecules [49], further highlighting its potential to act as a model geophyte species, thus warranting further investigation.

Concentrations greater than 1.0 mg/L were found to be cytotoxic, resulting in fragment bleaching and consequential death. This finding is consistent with those of Mohanpuria and Sadav [52], which demonstrated that a concentration of caffeine (1 mM) adversely affected the development of *Arabidopsis thaliana* and tobacco seedlings. Furthermore, the same study by Mohanpuria and Sadav showed that at a higher concentration of 5 mM, both species exhibited chlorosis and early senescence, effects that were linked to a negative impact on Rubisco activity [52]. The ability of caffeine to stimulate cellular growth is noteworthy, suggesting a hormone-like activity that may be linked to its structural affinity to cytokinin and auxin. In fact, when caffeine was used in combination with IAA and TDZ, the rate of fragments’ response increased to nearly 100%, indicating either a synergistic or additive effect; however, it is currently not possible to differentiate between the two scenarios. L-Kynurenine, an IAA inhibitor, did not clarify the potential link between caffeine and its mode of action. This lack of inhibition may be attributed to the presence of the cytokinin TDZ in the same culture plates that contained the inhibitor. Indeed, TDZ has also been shown to induce callus formation [49], and at rates similar to those of IAA alone. However, the combination of the two resulted in better developed calli and micro-bulbs than either hormone alone, and alterations to these morphological characteristics were not observed in the combined treatment with IAA, TDZ, and caffeine. The lack of effects observed with the IAA inhibitor, L-Kynurenine, in the tissue culture of *Ornithogalum dubium* could also be explained by the possibility that IAA biosynthesis is ethylene-independent, as He *et al.,* demonstrated that L-Kynurenine acts as a competitive inhibitor of ethylene-directed auxin biosynthesis [53]. This current evidence, however, warrants further inhibitory studies.

The aforementioned findings are also consistent with those of Muratova and co-authors [30], who established the positive influence of caffeine (range 1 mg/L to 100 mg/L) on the rhizogenesis of *Rubus* species. Furthermore, when caffeine was combined with another hormone, 1- β-indolylbutyric acid (IBA) 1 mg/L, the rates of rhizogenesis were greater than when used on its own. Concentrations exceeding 100 mg/L were found to be detrimental, leading to necrosis and plant death. The negative impact of caffeine on adventitious root formation was also observed in hypocotyl cuttings of mung bean (*Phaseolus aureus*) when tested at the concentration of 1 mM, resulting in reduced root formation and complete inhibition at 2 mM [54]. The study concluded that impairment in protein biosynthesis and a negative effect on polyphenol oxidase, PPO, were potential factors contributing to the adverse effects of caffeine on rhizogenesis. Additionally, Smyth [55] reported that roots exhibited greater sensitivity to caffeine when applied to rice seedlings at a concentration of 2.5 mM, resulting in a 90% reduction in root length observed 6 days post-exposure.

Caffeine has been reported to be both cytotoxic in various systems, including human cells, and mutagenic with the ability to induce chromosomal endoduplication and, consequently, polyploidization [56,57,58,59,60]. In this study, we observed no alterations in chromosomes at the concentration effective for inducing cellular replication and re-differentiation (0.025 mg/L, Figure 7). Compared to other studies, this report used concentrations that were substantially lower than those previously reported. For instance, Roy [27] employed a concentration of 0.1% or equivalent to 1000 mg/L, which is approximately 10^4^-fold greater than the concentration used in this report (0.025 mg/L); however, Roy’s report showed some limited irregular duplication events [27]. Similarly, Broughton [29] established the optimal concentration in wheat at 0.1%, while Lim [28] reported an even higher optimal concentration of 0.3% in Lilium, which was 10^5^-fold larger. Duplication in wheat haploid lines was also achieved at the optimal concentration of 0.1% [61]. Based on this evidence, we can surmise that, as per other chemicals, caffeine can exert specific biological effects that are both concentration- and species-dependent. Further studies may be required in *Ornithogalum* to determine its heightened tissue sensitivity, as observed in this and other reports [49].

## 4. Materials and Methods

### 4.1. Plant Maintenance and Tissue Culture

*Ornithogalum dubium* plants were maintained in a temperature (22 ± 2 °C) and light (16 h light/8 h darkness) controlled glasshouse throughout the year. The plants were regularly watered and fertilized according to their needs. A commercial, peat-free compost was used for all stages of maintenance, amended with 1/3 of washed sand. Mature plants were employed for all stages of tissue culture. For the tissue culturing of *O. dubium*, leaves with a width of 1 to 2 cm were used, and a completely randomized approach was used throughout the collection of leaves. For sterilization purposes, the protocol described in [49] was used without modifications.

### 4.2. Chemical Stocks

All chemicals used throughout the tissue culture experiments were obtained from Duchefa (Haarlem, The Netherlands) and were dissolved in Ethanol (Indole acetic Acid), DMSO (Thiaurazon and L-kynurenine), or water (caffeine), filter-sterilized, and maintained as stock at −20 °C.

### 4.3. Media Preparation

MS basal salts + 3% sucrose pH 5.7 medium was used throughout all the experiments. Hormones (IAA, TDZ) and other chemicals (CAF and L-kynurenine) were added to the medium once it had cooled down to 55 °C. Caffeine was also added to the medium at the same temperature despite being thermostable.

### 4.4. Evaluation of Caffeine Concentrations on O. dubium Regeneration Potentials

For these sets of experiments, caffeine on its own was used at either low concentrations (0.0125/0.025/0.05/0.0625 mg/L) or high concentrations (1/1.5/2.0 mg/L). Each experiment included 10/20 plates with 6/8 fragments of similar sizes (0.5 cm in thickness and 1/3 cm in length), with an average across experiments of 7 fragments. Each experiment was triplicated at 3-to-4-month intervals. The total number of fragments per treatment varied between 70 and 140. Plant material was incubated at 22 ± 2 °C in complete darkness. Plates that were contaminated were removed and not included in the rate and percentage calculations. Plates were regularly monitored for callus formation and/or micro bulbil development. Once these were approx. 0.5 cm in length/diameter or greater, plates were transferred to the glasshouse and maintained in a sealed transparent plastic box until chlorophyll development took place. Fragments containing clusters of plantlets/bulbils were removed from the agar and implanted in a 50%/50% peat-less compost and sand mixture. Plants were not watered for 2/3 days to prevent fungal infections. Rooted and rootless fragments were then watered from below, maintaining the moisture content to a minimum. Once evidence of growth was visible, watering was adjusted as required. When plantlets were of a manageable size (7/10 cm in height), they were transferred to individual pots (5 cm × 10 cm) either as independent plants or as clumps and grown to maturity.

### 4.5. Evaluation of Caffeine Effect on Treatments with Phytohormones

A secondary set of experiments was set up to compare the optimal caffeine-induced rates of regeneration to that of IAA+TDZ (0.5/0.5 mg/L) and the combination of IAA+TDZ and CAF (0.025 mg/L). The experiments included 3 replicates, each consisting of 20 Petri dishes, with each Petri dish containing 6 to 8 fragments of similar sizes (0.5 cm in height by 1–2 cm in length), with an average of 7 fragments per dish across experiments. The total number of fragments per treatment averaged 140. Plant material was incubated at 22 ± 2 °C in complete darkness. Contaminated plates were removed and excluded from the rate and percentage calculations. Plates were regularly monitored for callus formation, plantlet development, and/or micro bulbil formation. Once these were approx. 0.5 cm in length/diameter, or greater, plates were transferred to the glasshouse until chlorophyll development took place. Fragments containing clusters of plantlets/bulbils were removed from the agar and implanted in a 50%/50% peat-free compost and sand mixture.

### 4.6. Evaluation of Indole Acetic Acid Inhibitor

To assess the link between caffeine and its auxin-like activity, an IAA inhibitor was used. This was L-kynurenine [53], used at the following concentrations ranging from 0.1 mg/L to 2.0 mg/L. The treatments included L-kynurenine on its own (0./0.4/0.5/0.6/1.0 mg/L) and in combination with IAA+TDZ (0.5/0.5/0.5 or 1.0 or 2.0 mg/L) and CAF (0.025/0.5 mg/L). The experiments included 3 replicates with 20 Petri dishes, each containing 6 to 8 fragments of similar sizes (0.5 cm in height by 1–2 cm in length), resulting in an average of 7 fragments across experiments. The total number of fragments per treatment averaged 140. The plant material was incubated at 22 ± 2 °C in complete darkness.

### 4.7. Morphological Evaluation

Ten independent plants regenerated from 3 independent tissue culture experiments were used to morphologically characterize caffeine, control-derived, and Indole-acetic acid(IAA)/Thidiazuron (TDZ)-derived plants. The parameters assessed included leaf and flower morphology, pollen shape and viability, as well as tuber size and overall morphology.

Cytological investigations included leaf surface characteristics, leaf sections, and chromosomal counting. To evaluate guard cells and stomata, a minimum of 3 leaves were used for the caffeine and AUX+TDZ treatments. Adaxial and abaxial surfaces were observed under 20× and 40× magnification (Leica, Wetzlar, Germany) without the application of any additional dye. Same-size leaves were used for the comparison. For chromosomal counting, mitotic cells arising from ongoing tissue culture experiments were used along with acetocarmine and standard staining protocol. Small calli, between 1 and 3 mm in diameter, originating from independent plates were used for the CAF and IAA+TDZ treatments. At least 20 independent squashes from 5 sets of plates were used.

### 4.8. Statistical Analysis

Statistical analyses were performed using SPSS 27 (IBM, New York, NY, USA). All experiments were examined for normality (Kolmogorov–Smirnov test) and examined by ANOVA with post hoc for parametric normally distributed data (bulb size) or in non-parametric cases (proportion rates and regeneration percentage) by Kruskal–Wallis. Significance was established at the alpha value of 0.05 or lower.

## 5. Conclusions

Caffeine is an interesting molecule that has a significant impact on humans. While extensively studied for its effects on human health, research on caffeine in plant science has been limited, primarily focusing on its well-established antimicrobial and insect repellent properties. Very few studies have been carried out on caffeine in the context of tissue culture. In this report, we demonstrate that caffeine can stimulate cellular dedifferentiation and redifferentiation at low concentrations (0.025 mg/L) in the highly receptive plant species *Ornithogalum dubium*. This study is one of the few reports that indicated the ability of caffeine to stimulate cellular growth in both an unspecific manner (callus formation) and a specific manner (direct organogenesis). It was also found that the effective concentration range for *O. dubium* is narrow, limiting its applicability. It was also shown that no alterations in chromosome numbers were detected, and the general morphological characteristics of the regenerated plants were comparable to those produced using canonical hormones, such as IAA. These findings add caffeine to the toolbox of plant scientists but warrant further investigation into caffeine’s potential mechanisms of action and, more importantly, its applicability to other plant systems.

## Data Availability

Data are available from the author upon request.

## Supplementary Materials

The following supporting information can be downloaded at: https://www.mdpi.com/article/10.3390/plants14071127/s1, Figure S1: Developmental differential between Caffeine and IAA+TDZ—induced callus after 1 month of culture.; Figure S2: Cytotoxic effect of Caffeine at higher concentrations (1.0/1.5 and 2.0 mg/L), as determined 4 weeks post-induction. Table S1: Kynurenine treatments on tissue cultures of *Ornithogalum dubium*.

## References

[1] Ashihara H., Suzuki T. (2004). Distribution and biosynthesis of caffeine in plants. Front. Biosci..

[2] Kretschmar J.A., Baumann T.W. (1999). Caffeine in Citrus flowers. Phytochemistry.

[3] Ashihara H., Crozier A. (2001). Caffeine: A well known but little mentioned compound in plant science. Trends Plant Sci..

[4] Hewavitharanage P., Karunaratne S., Kumar N.S. (1999). Effect of caffeine on shot-hole borer beetle (Xyleborusfornicatus) of tea (*Camellia sinensis*). Phytochemistry.

[5] Guerreiro Filho O., Mazzafera P. (2003). Caffeine and resistance of coffee to the berry borer *Hypothenemus hampei* (Coleoptera: Scolytidae). J. Agric. Food Chem..

[6] Phankaen Y., Manaprasertsak A., Pluempanupat W., Koul O., Kainoh Y., Bullangpoti V. (2017). Toxicity and repellent action of Coffea arabica against Tribolium castaneum (Herbst) adults under laboratory conditions. J. Stored Prod. Res..

[7] Jurić S., Vinceković M., Marijan M., Vlahoviček-Kahlina K., Galešić M.A., Orešković M., Lemic D., Čirjak D., Pajač Živković I. (2023). Effectiveness of aqueous coffee extract and caffeine in controlling phytophagous heteropteran species. Appl. Ecol. Environ. Res..

[8] Chou C.H., Waller G.R. (1980). Possible allelopathic constituents of Coffea arabica. J. Chem. Ecol..

[9] Waller G.R. (1989). Biochemical frontiers of allelopathy. Biol. Plant..

[10] Silva R.M., Brigatti J.G., Santos V.H., Mecina G.F., Silva L.P. (2013). Allelopathic effect of the peel of coffee fruit. Sci. Hortic..

[11] Tanti A., Bhattacharyya P.N., Sandilya S.P., Dutta P. (2016). Allelopathic potential of caffeine as growth and germination inhibitor to popular tea weed, *Boreria hispida* L. Curr. Life Sci..

[12] Pham V.T.T., Ismail T., Mishyna M., Appiah K.S., Oikawa Y., Fujii Y. (2019). Caffeine: The allelochemical responsible for the plant growth inhibitory activity of Vietnamese tea (*Camellia sinensis* L. Kuntze). Agronomy.

[13] da Rosa S.D.V.F., de Oliveira Vilela A.L., Alves M.V.P., Cardoso M.D.G., Vieira L.F.A., Ferreira A.M.O., da Silva L.M. (2025). Allelopathic activity of coffee extracts: Implications for germination and initial growth in select weeds and polyploidy in *Lactuca sativa* L. J. Toxicol. Environ. Health Part A.

[14] Lin Z., Wei J., Hu Y., Pi D., Jiang M., Lang T. (2023). Caffeine synthesis and its mechanism and application by microbial degradation, A review. Foods.

[15] Huang R., O’Donnell A.J., Barboline J.J., Barkman T.J. (2016). Convergent evolution of caffeine in plants by co-option of exapted ancestral enzymes. Proc. Natl. Acad. Sci. USA.

[16] Temple J.L., Bernard C., Lipshultz S.E., Czachor J.D., Westphal J.A., Mestre M.A. (2017). The safety of ingested caffeine: A comprehensive review. Front. Psychiatry.

[17] Wikoff D., Welsh B.T., Henderson R., Brorby G.P., Britt J., Myers E., Goldberger J., Lieberman H.R., O’Brien C., Peck J. (2017). Systematic review of the potential adverse effects of caffeine consumption in healthy adults, pregnant women, adolescents, and children. Food Chem. Toxicol..

[18] Camargo M.A.F., Camargo C.A.C.M. (2019). Effects of Caffeine on the Organism—Literature Review. Open Access Libr. J..

[19] Ibrahim S.A., Salameh M.M., Phetsomphou S., Yang H., Seo C.W. (2006). Application of caffeine, 1, 3, 7-trimethylxanthine, to control Escherichia coli O157: H7. Food Chem..

[20] Lele O.H., Maniar J.A., Chakravorty R.L., Vaidya S.P., Chowdhary A.S. (2016). Assessment of biological activities of caffeine. Int. J. Curr. Microbiol. Appl. Sci..

[21] Sledz W., Los E., Paczek A., Rischka J., Motyka A., Zoledowska S., Piosik J., Lojkowska E. (2015). Antibacterial activity of caffeine against plant pathogenic bacteria. Acta Biochim. Pol..

[22] Mohammadi S., Maghsoudloo M., Maroufi M. (2023). Antioxidant, antineurodegenerative, anticancer, and antimicrobial activities of caffeine and its derivatives: Micro and nano aspects. Micro Nano Bio Asp..

[23] Kim Y.S., Uefuji H., Ogita S., Sano H. (2006). Transgenic tobacco plants producing caffeine: A potential new strategy for insect pest control. Transgenic Res..

[24] Kim Y.S., Sano H. (2008). Pathogen resistance of transgenic tobacco plants producing caffeine. Phytochemistry.

[25] Kim Y.S., Choi Y.E., Sano H. (2010). Plant vaccination: Stimulation of defense system by caffeine production in planta. Plant Signal. Behav..

[26] Sano H., Kim Y.S., Choi Y.E. (2013). Like cures like: Caffeine immunizes plants against biotic stresses. Advances in Botanical Research.

[27] Roy S.C. (1973). Comparative effects of colchicine, caffeine. Biol. Plant..

[28] Lim K.B., Gonzalez R.B., Zhou S., Ramanna M.S., Van Tuyl J.M. (2005). Meiotic polyploidization with homoeologous recombination induced by caffeine treatment in interspecific lily hybrids. Korean J. Genet..

[29] Broughton S., Castello M., Liu L., Killen J., Hepworth A., O’Leary R. (2020). The effect of caffeine and trifluralin on chromosome doubling in wheat anther culture. Plants.

[30] Muratova S.A., Papikhin R.V., Khoroshkova Y.V. (2020). The effect of caffeine in a nutrient medium on rhizogenesis of the Rubus genus plants. BIO Web of Conferences.

[31] Manning J.C., Forest F., Devey D.S., Fay M.F., Goldblatt P. (2009). A molecular phylogeny and a revised classification of Ornithogaloideae (Hyacinthaceae) based on an analysis of four plastid DNA regions. Taxon.

[32] Reinten E.Y., Coetzee J.H., Van Wyk B.E. (2011). The potential of South African indigenous plants for the international cut flower trade. S. Afr. J. Bot..

[33] Griesbach R.J., Meyer F., Koopowitz H. (1993). Creation of new flower colors in Ornithogalum via interspecific hybridization. J. Am. Soc. Hortic. Sci..

[34] Littlejohn G.M. (2007). Star of Bethlehem: Ornithogalum. Flower Breeding and Genetics: Issues, Challenges and Opportunities for the 21st Century.

[35] Luria G., Watad A.A., Cohen-Zhedek Y., Borochov A. Growth and flowering of Ornithogalum dubium. Proceedings of the VIII International Symposium on Flowerbulbs.

[36] Niederwieser J.G., Van De Venter H.A., Robbertse P.J. (1990). Embryo rescue in Ornithogalum. HortScience.

[37] Lee J., Gómez M.I., Miller W.B. (2015). Paclobutrazol and flurprimidol control stem elongation of potted star of Bethlehem. HortTechnology.

[38] Lee J., Miller W.B. (2015). Preplant storage and greenhouse temperature influence flowering of Ornithogalum. HortScience.

[39] Joshi J.R., Yedidia I. Breeding for resistance to soft rot disease in Ornithogalum. Proceedings of the XII International Symposium on Flower Bulbs and Herbaceous Perennials.

[40] Lipsky A., Joshi J.R., Carmi N., Yedidia I. (2016). Expression levels of antimicrobial peptide tachyplesin I in transgenic Ornithogalum lines affect the resistance to Pectobacterium infection. J. Biotechnol..

[41] De Villiers S.M., Kamo K., Thomson J.A., Bornman C.H., Berger D.K. (2000). Biolistic transformation of chincherinchee (Ornithogalum) and regeneration of transgenic plants. Physiol. Plant..

[42] Cohen A., Lipsky A., Arazi T., Ion A., Stav R., Sandler-Ziv D., Fintea C., Gaba V., Gera A. Particle bombardment-mediated transformation of Ornithogalum dubium for Ornithogalum mosaic virus resistance. Proceedings of the IX International Symposium on Flower Bulbs.

[43] Van Emmenes L., Veale A., Cohen A., Arazi T. Agrobacterium-mediated transformation of the bulbous flower Ornithogalum. Proceedings of the XXVII International Horticultural Congress-IHC2006: International Symposium on Ornamentals, Now!.

[44] Tripathi P.K., Ayzenshtat D., Kumar M., Zemach H., Yedidia I., Bocobza S.E. (2023). An efficient and reproducible Agrobacterium-mediated genetic transformation method for the ornamental monocotyledonous plant Ornithogalum dubium Houtt. Plant Growth Regul..

[45] Ziv M., Lilien-Kipnis H. (2000). Bud regeneration from inflorescence explants for rapid propagation of geophytes in vitro. Plant Cell Rep..

[46] Malabadi R.B., Van Staden J., Bornman C.H. (2004). Regeneration of Ornithogalum in vitro. S. Afr. J. Bot..

[47] López-Marín J., González A., Cos J. In Vitro Multiplication of Four Species of the Genus Ornithogalum. Proceedings of the III International Symposium on Acclimatization and Establishment of Micropropagated Plants.

[48] Suh J.K., Lee W.H., Lee A.K. New plantlet proliferation and bulbing promotion in in vitro culture of Ornithogalum hybrid. Proceedings of the V International Symposium on New Floricultural Crops.

[49] Petti C. (2020). Phloroglucinol Mediated Plant Regeneration of Ornithogalum dubium as the Sole “Hormone-Like Supplement” in Plant Tissue Culture Long-Term Experiments. Plants.

[50] Tun O.M., Lipsky A., Luzzatto Knaan T., Kerem Z., Yedidia I. (2013). The plant activator BTH promotes *Ornithogalum dubium* and *O. thyrsoides* differentiation and regeneration in vitro. Biol. Plant..

[51] da Silva J.A.T., Dobránszki J., Ross S. (2013). Phloroglucinol in plant tissue culture. In Vitro Cell. Dev. Biology. Plant.

[52] Mohanpuria P., Yadav S.K. (2009). Retardation in seedling growth and induction of early senescence in plants upon caffeine exposure is related to its negative effect on Rubisco. Photosynthetica.

[53] He W., Brumos J., Li H., Ji Y., Ke M., Gong X., Zeng Q., Li W., Zhang X., An F. (2011). A small-molecule screen identifies L-kynurenine as a competitive inhibitor of TAA1/TAR activity in ethylene-directed auxin biosynthesis and root growth in Arabidopsis. Plant Cell.

[54] Batish D.R., Singh H.P., Kaur M., Kohli R.K., Yadav S.S. (2008). Caffeine affects adventitious rooting and causes biochemical changes in the hypocotyl cuttings of mung bean (Phaseolus aureus Roxb.). Acta Physiol. Plant..

[55] Smyth D.A. (1992). Effect of methylxanthine treatment on rice seedling growth. J. Plant Growth Regul..

[56] Sivak A., Rudenko L., Teague L.G. (1982). Variations among species and cell types in the effects of caffeine on mutagen-induced cytotoxicity and postreplication repair of DNA. Environ. Mutagen..

[57] Gonzalez-Fernandez A., Hernandez P., Lopez-Saez J.F. (1985). Effect of caffeine and adenosine on G2 repair: Mitotic delay and chromosome damage. Mutat. Res./Fundam. Mol. Mech. Mutagen..

[58] Lock R.B., Galperina O.V., Feldhoff R.C., Rhodes L.J. (1994). Concentration-dependent differences in the mechanisms by which caffeine potentiates etoposide cytotoxicity in HeLa cells. Cancer Res..

[59] Truţă E., Zamfirache M.M., Olteanu Z. (2011). Caffeine induced genotoxic effects in *Phaseolus vulgaris* L. and *Raphanus sativus* L. Bot. Serbica.

[60] Chung W.H. (2020). Pleiotropic effects of caffeine leading to chromosome instability and cytotoxicity in eukaryotic microorganisms. J. Microbiol. Biotechnol..

[61] Thomas J., Chen Q., Howes N. (1997). Chromosome doubling of haploids of common wheat with caffeine. Genome.

