# Supplementary material for: Low Caffeine Concentrations Induce Callus and Direct Organogenesis in Tissue Cultures of Ornithogalum dubium
Source: Plants (Basel). 2025 Apr 5;14(7):1127. doi: 10.3390/plants14071127 (PMC11991515; doi:10.3390/plants14071127)
Supplement: Supplementary file 1 [file plants-14-01127-s001.zip › plants-3515401-supplementary.pdf]

Supplementary material

This file provides additional material as presented in the manuscript and it is made of 2 Supplementary Figures and a supplementary Table S1.

Figures.

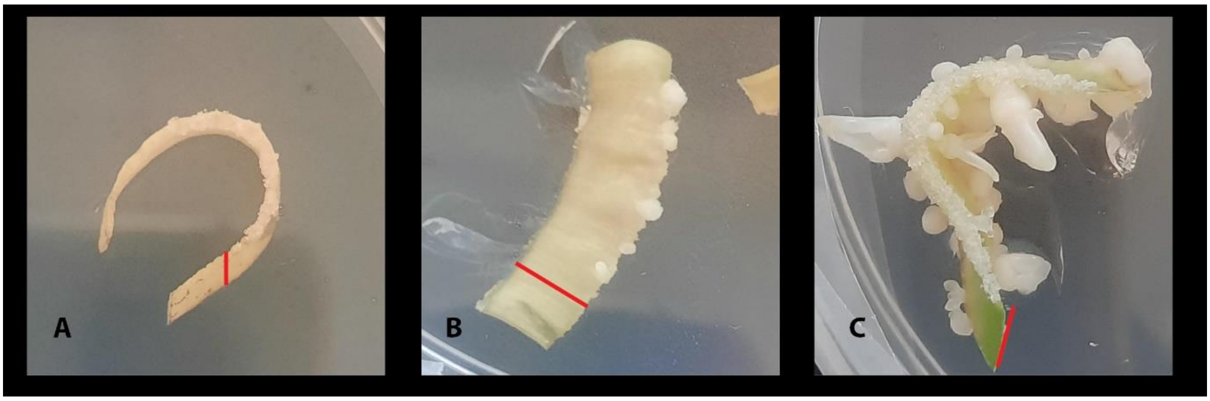

Supplementary Figure S1. Developmental differential between Caffeine and IAA+TDZ -induced callus after 1 month of culture. Calli appeared more prominent and well developed in the hormone treatment (IAA+TDZ, C), when compared to CAF (B) and control (A). Caffeine however also showed a more pronounced effect when compared to that of the control (no chemicals).

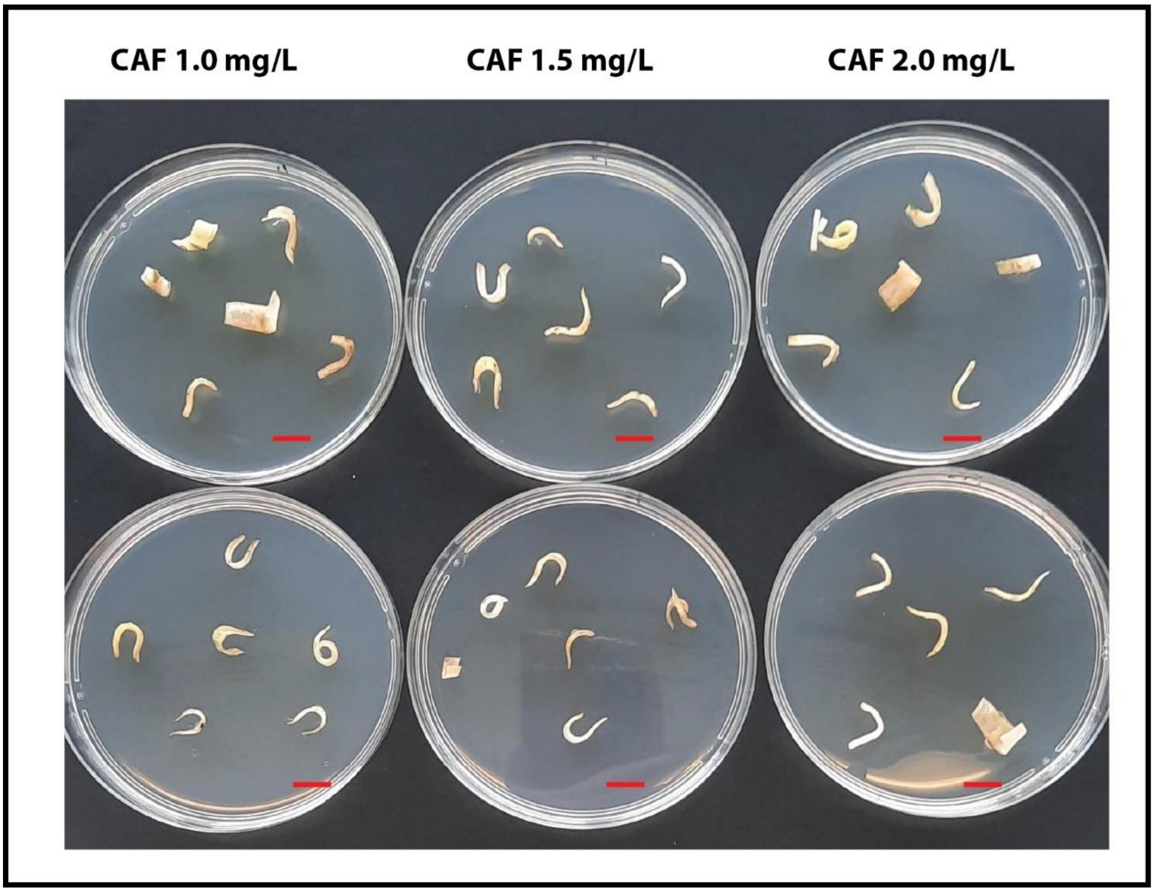

Supplementary Figure S2. Cytotoxic effect of Caffeine at higher concentrations (1.0/1.5 and 2.0 mg/L), as determined 4 weeks post-induction. Red bar equates to 1 cm.

Supplementary Table S1. Kynurenine treatments on tissue cultures of *Ornithogalum dubium*. Kynurenine was used in absence of hormones in various rates and in combination with IAA+TDZ and CAF. Same letters indicate no significant differences (P>0.05). Different letters indicate a significant difference at the α-value of 0.05.

Treatment	Concentration of Compound (mg/L)	Regeneration rates (%)	Observations
Control	0	14.3 +/- 7.91a	Chlorotic fragments
L-kynurenine	0.4	15.2 +/- 5.23a	Green fragments
L-kynurenine	0.5	16.3 +/- 6.23a	Green fragments
L-kynurenine	0.6	16.8 +/- 4.72a	Green fragments
L-kynurenine	1.0	17.2 +/- 6.84a	Green fragments
*IAA+TDZ+L-Kyn	0.5/0.5+0.5	91.0 +/- 5.93b	Green fragments
*IAA+TDZ+L-Kyn	0.5/0.5+1.0	93.2 +/- 6.52b	Green fragments
*IAA+TDZ+L-Kyn	0.5/0.5+2.0	89.3 +/-8.61b	Green fragments
**CAF+***L-Kyn	0.025+0.5	49.8 +/- 7.25c	Green fragments

*IAA: Indole Acetic Acid, TDZ; Thiadurazon

**CAF: Caffeine

*** L-kynurenine
